# Eucalyptus Tree

**Published:** 1878-05

**Authors:** 


EUCALYPTUS-TREE.—The anti-malarial action of the eucalyptus-tree is called in question by Mr. Arthur Nichols, who, writing in *Nature*, says that in Queensland, in the very heart of a forest extending for many miles in every direction, and composed mainly of eucalyptus of every variety, he has himself suffered from malaria, and has known many instances of febrile attacks among shepherds and stockmen in the locality. On inquiry, he learned that these attacks were not confined to any particular year, but that every year some cases might be expected. Again, it has been asserted that, wherever the eucalyptus had been introduced on a considerable scale in Algeria, the mosquitoes all disappeared. But this correspondent, writing of Australia, says that he has found these pests so intolerable on high land, where almost the only variety of tree to be found was one variety or another of eucalyptus, and sometimes all, that sleep was impossible while camping out at night, life a burden during the day, by reason of these insects.

The anti-malarial properties of the *Eucalyptus globulus* are commonly supposed to depend exclusively on the emanations from the leaves; but Mr. A. W. Bennett thinks it most probable that the chief effect is produced by the action of the roots on the soil. Writing of this subject in *Nature*, he remarks that the effect of the planting of forests in increasing the rainfall is often erroneously reputed to be due to the "attractive force of the trees" on the moisture in the air, similar to that exerted by a range of mountains; but this supposition he regards as untenable. The mode in which trees mainly act is, he says, by their roots arresting the rainfall which would otherwise escape by the natural drainage of the country; the combined forces of capillarity, osmose, and transpiration, then cause the ascent through the tissues of the tree of the water thus arrested, and the larger portion is eventually given off into the air through the stomata of the leaves. In this way a forest-tree will in a very short time give off into the air its own weight of water, which is again deposited as rain or dew. It is quite possible, however, that the effect of the planting of trees may be *apparently* the reverse of this in swampy regions without natural drainage. The water then accumulates in the soil; and if the country is bare of timber-trees and the sun powerful, a rapid decomposition takes place of the herbaceous vegetation, with consequent emanation of malarial vapors. If trees be planted, the effect is to supply natural drainage; the accumulation of water in the soil, and the consequent noxious effluvia, will be diminished and finally prevented, and the atmosphere rendered, if not drier, at least more wholesome.—*Popular Science Monthly.—Supplement.*

